# Pre-operative intradermal administration of CpG-B ± GM-CSF in stage I-III melanoma patients arms the sentinel lymph node: evidence for reduced tumor spread

**DOI:** 10.1186/2051-1426-1-S1-P92

**Published:** 2013-11-07

**Authors:** Mari F van den Hout, Bas D Koster, Rik J Scheper, Rieneke van de Ven, Berbel J Sluijter, Barbara G Molenkamp, Alfons J van den Eertwegh, Paul A van Leeuwen, Petrousjka M van den Tol, Tanja D de Gruijl

**Affiliations:** 1 Medical Oncology, VU University Medical Center, Amsterdam, the Netherlands; 2 Pathology, VU University Medical Center, Amsterdam, the Netherlands; 3 Surgical Oncology, VU University Medical Center, Amsterdam, the Netherlands

## 

Melanoma cutis is virtually incurable when it has metastasized to distant organs. Unfortunately, there is no safe and effective adjuvant treatment available for early stage patients that may prevent the development of metastasis. Since early melanoma development is accompanied by impaired immune effector functions in the sentinel lymph node (SLN), there is a strong rationale for therapeutic immune modulation of the SLN aimed at strengthening cellular immune functions. In two placebo controlled trials, the immunological effects of i.d. administration of CpG type-B (PF-3512676, 1 or 8 mg) ± GM-CSF (Leukine, 100 mg) at the tumor site, one week before SLN excision, were investigated. The trials showed that this treatment is clinically safe and flow cytometric and functional analyses of viable immune effector cells isolated from the SLN showed increased frequencies and activation state of LN resident CD141+ cDC subsets as well as activation of pDC and CD1a+ skin derived cDC subsets in the treated groups. This DC activation was accompanied by an increased Th1 cytokine response profile and melanoma-specific CD8+ T cell reactivity. Interestingly, when combining the treated groups of both studies (n=31), we found remarkable clinical differences compared to the combined placebo groups (n=22). Important predictors for recurrence and SLN tumor positivity are Breslow depth and ulceration of the primary tumor. These two clinical parameters were higher (although not significantly so) in the treated group. Nevertheless, the number of tumor positive SLN in this group was significantly lower (3/31 vs. 8/22 p=0.02). At a median follow-up of 71 months we also found a longer recurrence free survival in the treated group (p=0.14). We conclude that local administration of CpG ± GM-CSF is safe, shows very promising immunological efficacy with evidence for clinical impact on lymphatic spread and recurrence free survival and could potentially be used as adjuvant treatment combined with primary excision of suspected melanoma lesions.

